# Supplementary material for: Content Validity of a New Soccer (Football) Return-to-Play Test: The RONDO-TEST
Source: J Funct Morphol Kinesiol. 2024 Dec 25;10(1):3. doi: 10.3390/jfmk10010003 (PMC11755452; doi:10.3390/jfmk10010003)
Supplement: Supplementary file 1 [file jfmk-10-00003-s001.zip › File S1 - Procedures R1.pdf]

Journal of
Functional Morphology and Kinesiology

MDPI

File S1. Detailed description of RONDO-TEST execution

B1. Rondo Test: General implementation aspects

1. **Initial position:** The participant must stand (on both feet) 1 m behind the departure photoelectric beam break line facing the first diagonal.
2. **Execution:** The participant can start doing the circuit whenever they want, without giving any warning, taking the established route at top speed to complete the route as shown in the diagrams (see the test execution diagrams further below). The time taken will be measured to the accuracy of the hundredth of a second (0.01 s) and counted from the moment when the participant breaks the departure photoelectric beam to the moment when they break the same photoelectric beam upon completing the diagonals and finishing at the starting point. While doing the test, the participant is not allowed to knock over any of the obstacles (if they do, the test will be considered void and the participant will need to repeat it). If the participant does not do or gets one of the described tasks wrong, the attempt will be voided and they will need to repeat the entire test.
3. **Final position:** The test ends when the participant breaks the photoelectric beam after having completed the circuit.
4. **Examiner:** The examiner will monitor the execution of the test and will check the record on the computer to ensure it is correct.
5. **Venue:** The participant will do the test on the pitch where they play the most (the number of hours) throughout the season (natural or artificial turf pitch). The type of surface may affect the test results.
6. **Footwear:** To do the test, each player must wear their specific footwear (soccer boots with rubber or aluminium studs). The type of footwear may affect the test results.
7. **Recovery:** If a participant must repeat the test, they will be allowed a three-minute recovery period.

B2. Equipment positioning

A list of the equipment needed to do the test is shown below:

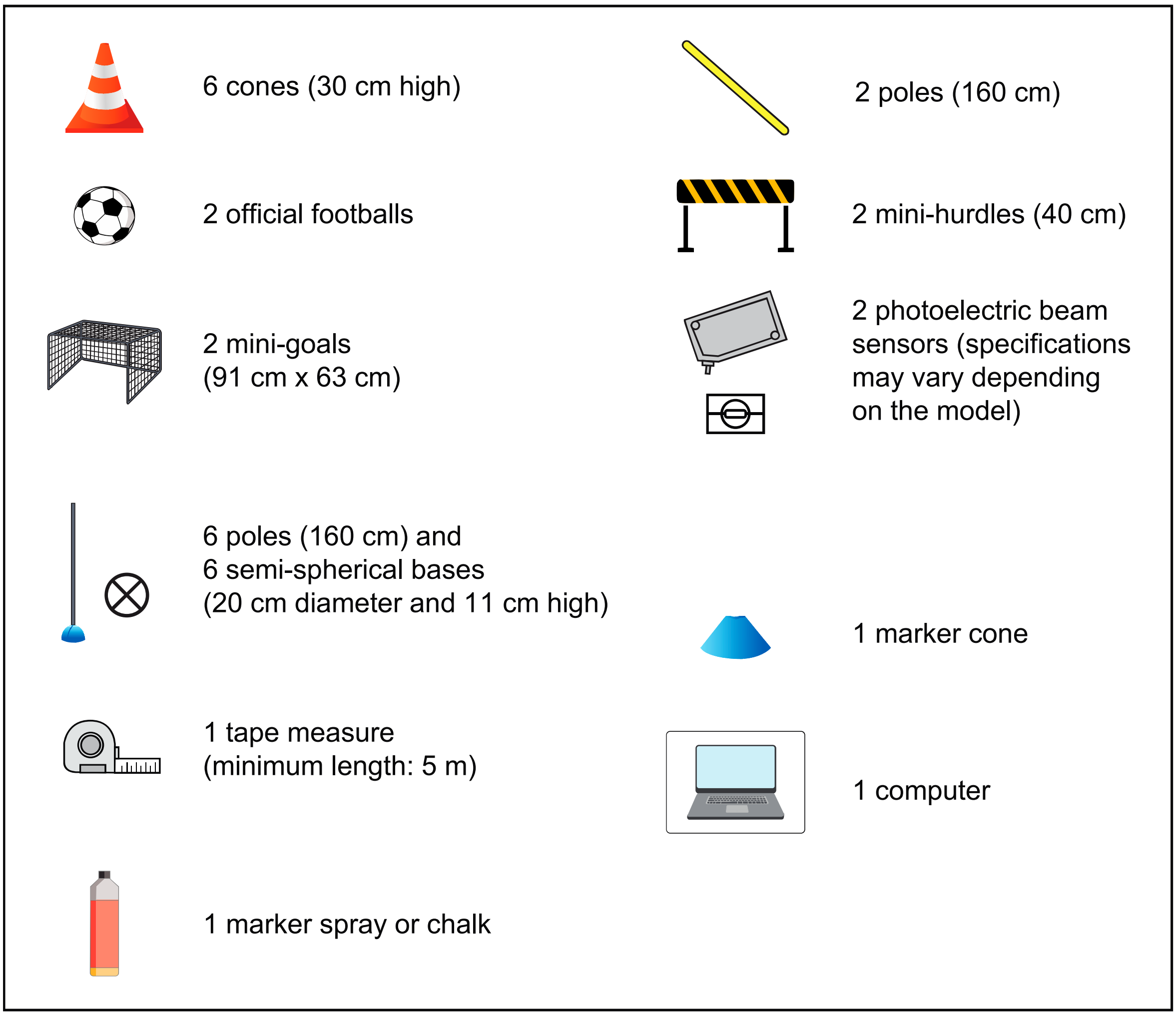

Regarding the criteria for equipment positioning, we would highlight the following:

1. A 30 cm diameter circular area must be marked out in the centre of the test circuit. The participant must pass through this area to activate and deactivate the photoelectric beam sensors.
2. A pole and its respective base must be positioned at the end of each diagonal (with one exception, as explained below) to force the participant to perform a turn. However, it is necessary to bear in mind that a marker cone must be placed on the first diagonal in Segment 2, instead of a pole with a base, because the participant will need to step on it.

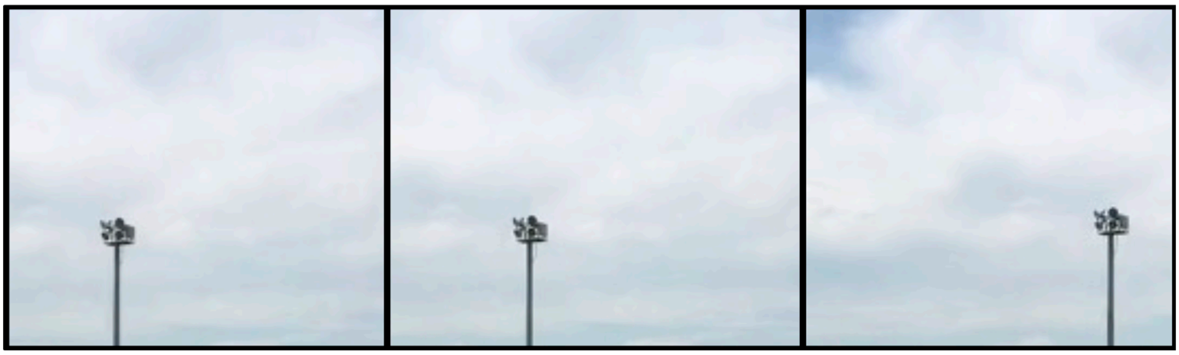

3. Each segment must be defined by a colour. All the equipment in a particular segment or diagonal must therefore be of the same colour. This will help to identify the actions that need to be performed on each of the diagonals throughout the route.

4. The cones marking the slalom and the zigzag must be positioned 0.5 m (on the horizontal axis) from the line and 1 m (on the vertical axis) apart (Segment 3).
5. A very important aspect is that it should be possible to knock over the 40 cm mini-hurdles in both directions of movement (Segment 4).
6. The first ball (the starting ball positioned in the centre of the circuit) must be placed 1 m from Segment 5, whereas the second ball must be positioned on the right-hand side (facing the mini-goal) and be touching the pole on the ground in Segment 7.
7. The distance and position of the photoelectric beam sensors are for the examiner to decide, since they may vary depending on the size of the tripods or the model of photoelectric beam sensors used. It is essential to observe the criterion that the photoelectric beam must be broken every time there is a change of segment. **Important!** The height at which the photoelectric beam is broken must be hip height. This is to ensure that it occurs in both photoelectric beam sensors at the same time in the centre of the test circuit.
8. The test ends at the point where it started.

B3. Graphical description of the equipment

B3.1 General equipment positioning (General Layout)

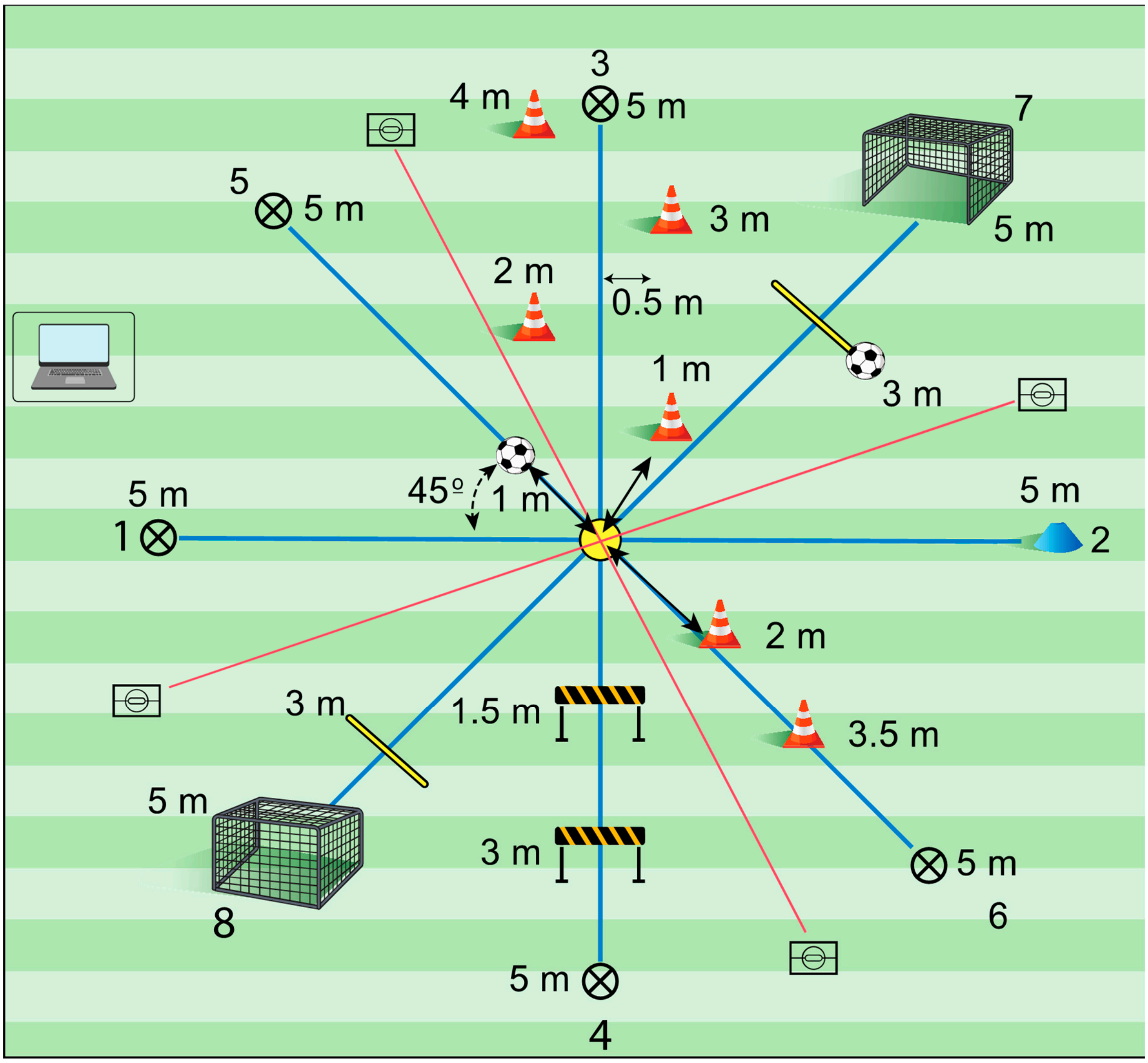

b) Equipment positioning on the first diagonal

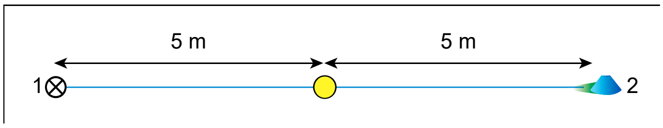

c) Equipment needed for the first diagonal (Segments 1 and 2)

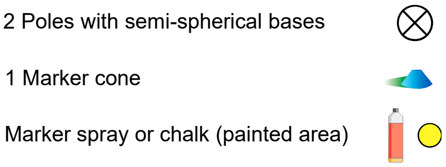

d) Equipment positioning on the second diagonal

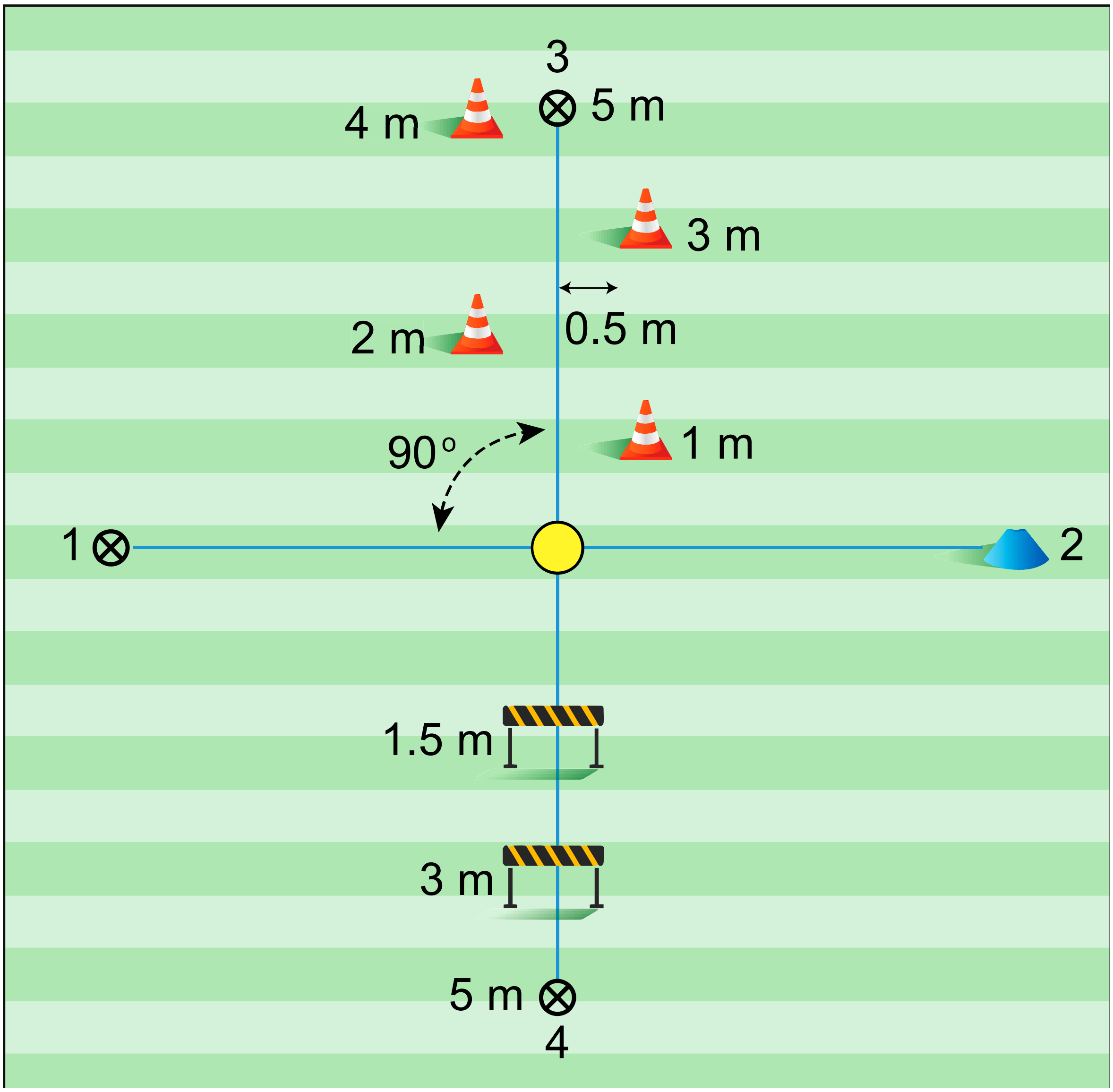

e) Equipment needed for the second diagonal (Segments 3 and 4)

2 Poles with semi-spherical bases

4 Cones

2 Mini-hurdles

f) Equipment positioning on the third diagonal

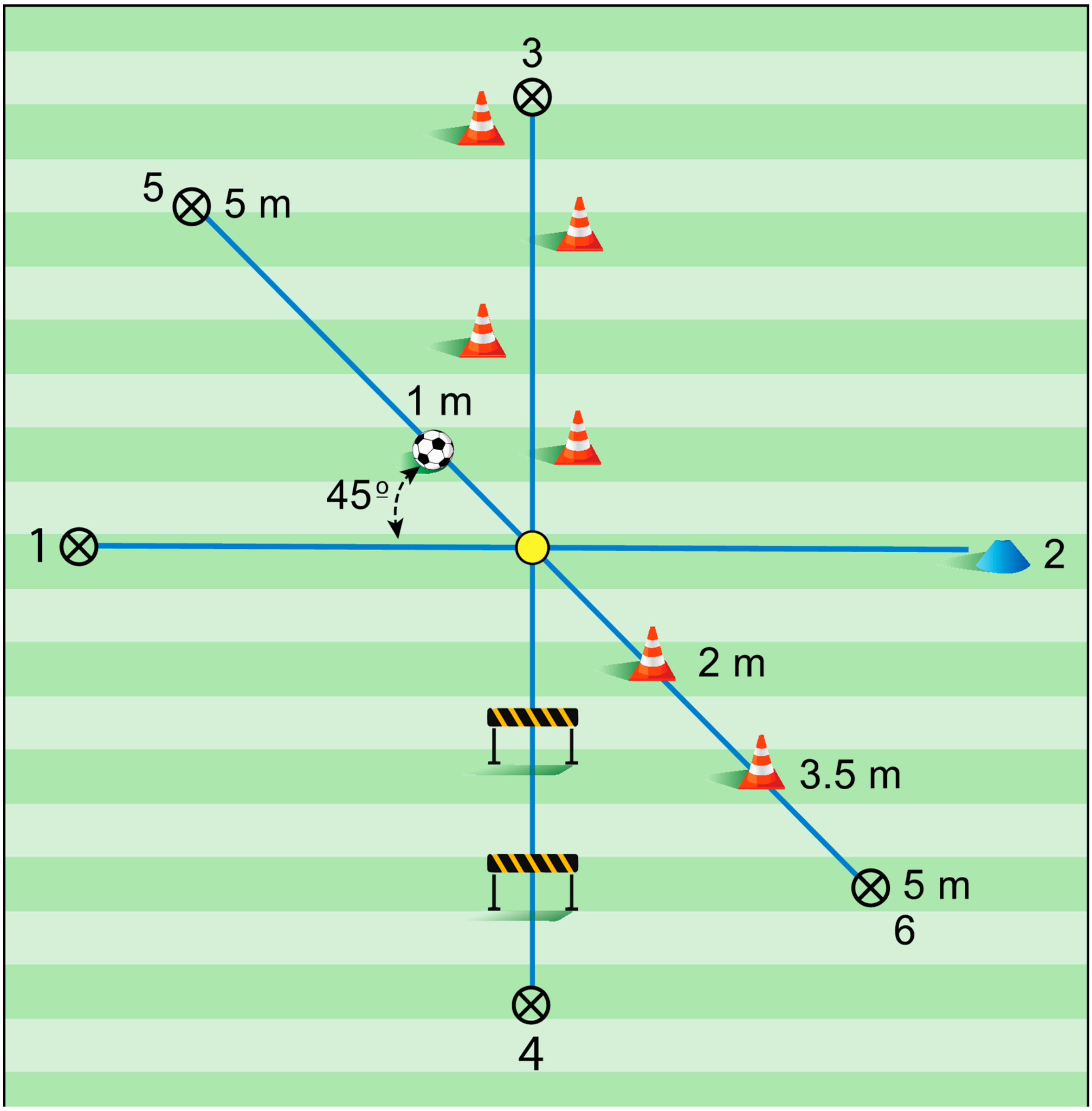

g) Equipment needed for the third diagonal (Segments 5 and 6)

2 Poles with semi-spherical bases

1 Football

2 Cones

h) Equipment positioning on the fourth diagonal

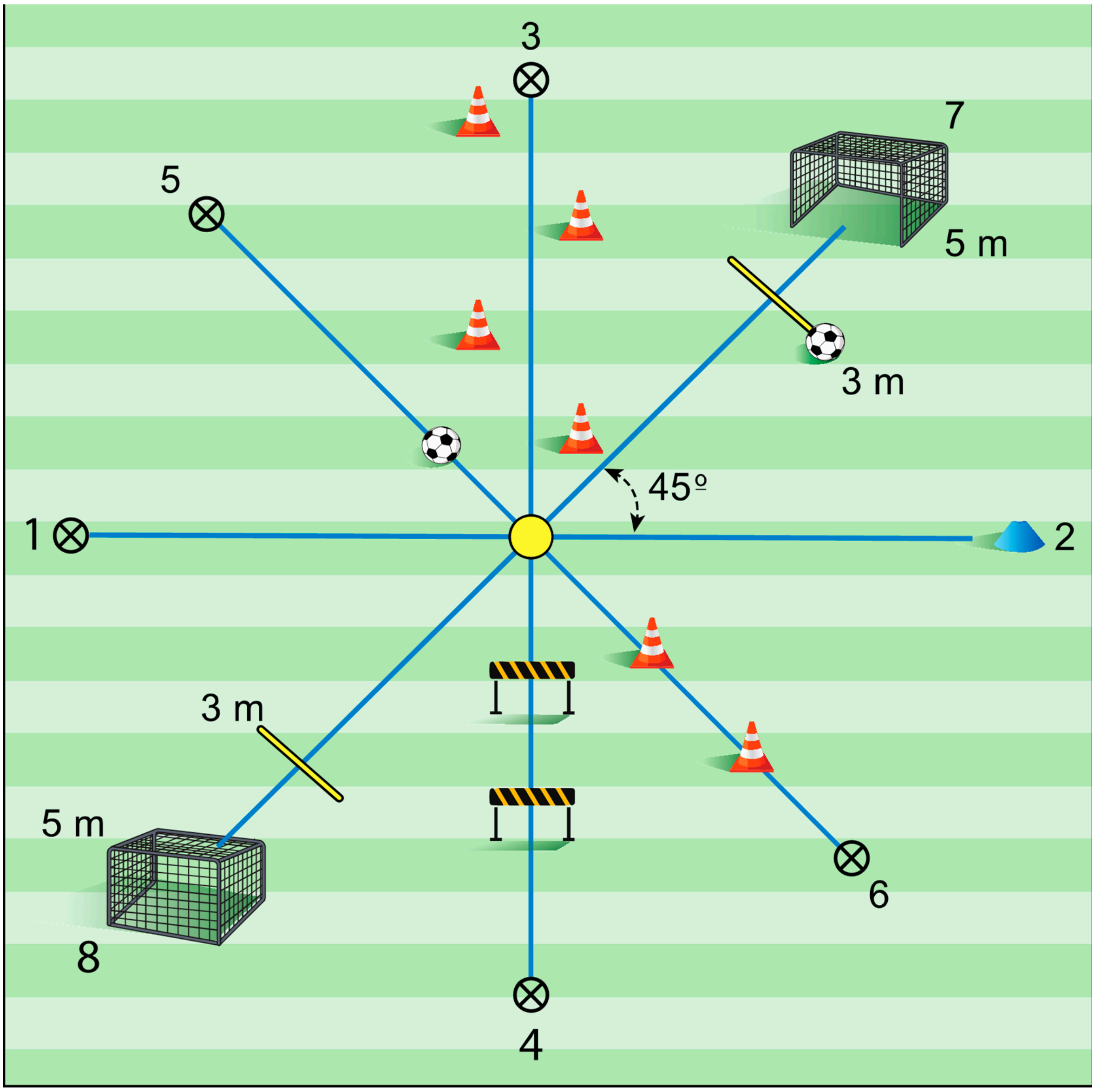

i) Equipment needed for the fourth diagonal (Segments 7 and 8)

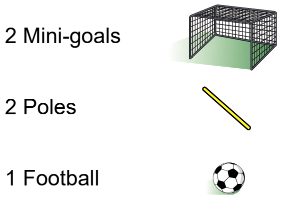

j) Positioning of the photoelectric beam sensors

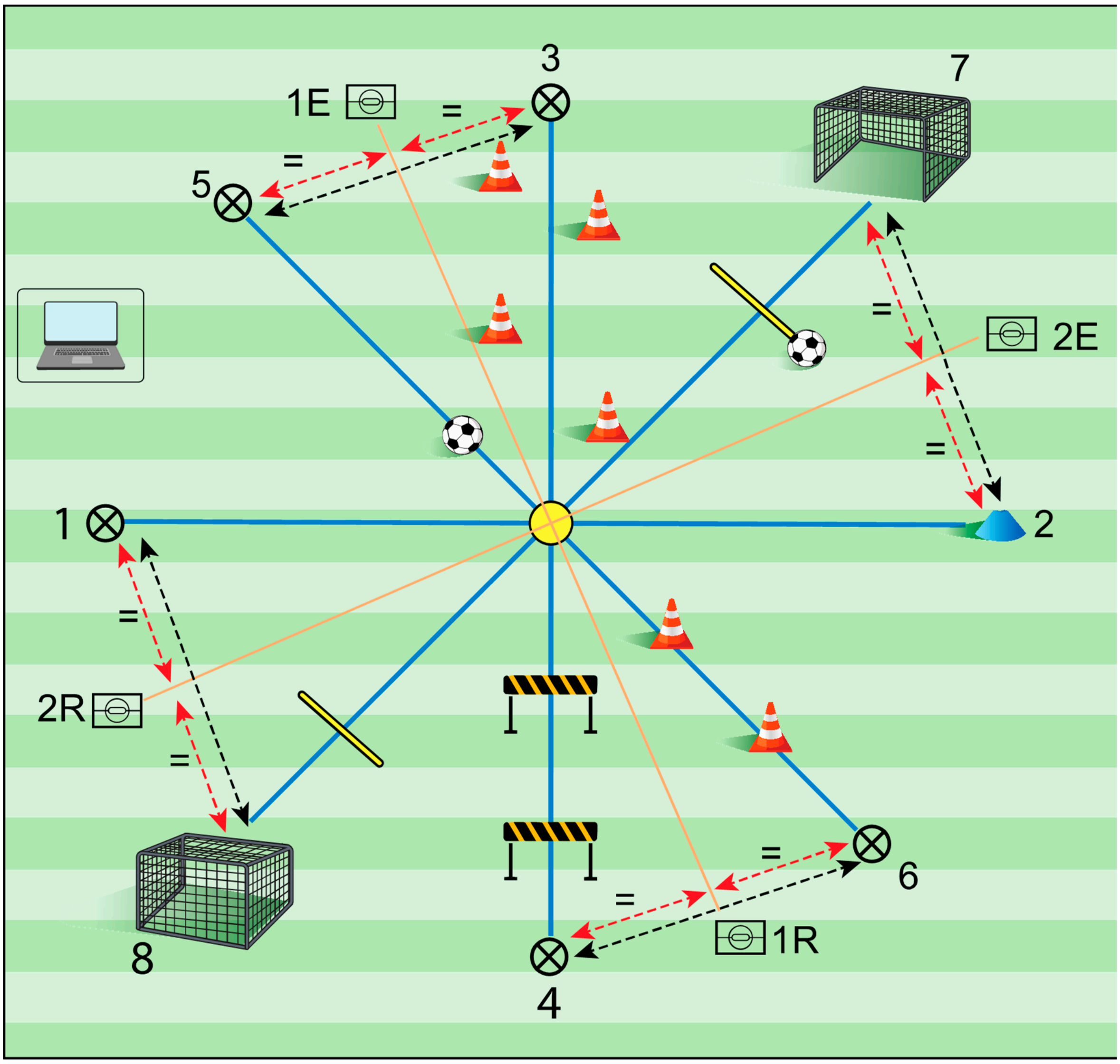

k) Equipment needed for the positioning of the photoelectric beam sensors.

Beam sensor 1	1 Emitter	1E
	1 Receiver	1R
Beam sensor 2	1 Emitter	2E
	1 Receiver	2R

B4. Test monitoring and follow-up recommendations

To ensure that the test is done correctly, the following aspects must be observed:

- Ensure that the turns are performed and executed correctly. The turns must always be done from outside to inside.
- Reinforce the test through verbal instructions from the tester and visual instructions from the colours in the segments.
- Ensure that there is a recovery period of at least 3–5 minutes between test series since it is estimated that the test can be done in approximately 30 seconds.
- Bear in mind that if any of the points listed below happen the test would be repeated (if any of them occur, the participant will be told to stop doing the test immediately

and be given the reason why; they will be given time to recover before repeating the test):

- The participant knocks over or moves the cones.
- The participant knocks over the mini-hurdles.
- The participant knocks over a pole when performing turns.
- The participant loses the ball.
- The participant does not score or hit the mini-goal.
- The participant does not perform the turns around the obstacles correctly.
- The participant does not touch the cones in the zigzags.
- The participant does not touch the marker cone in the sideways movement.
- The participant does not perform the jumps over the mini-hurdles correctly.
- The participant does not reach the pole when shooting the ball at the mini-goal.
- The participant touches any of the photoelectric beam sensors.
- There is a technological problem.

B5. 'Rondo Test': execution diagrams

First diagonal: The participant must stand 1 m away from the photoelectric beam break line. Facing forward, they must move forward to the first pole with a base and turn, continuing to move forward, but in the opposite direction towards the centre (Segment 1). From that moment, they must change orientation and move sideways to the marker cone, which they must touch, moving sideways again to return to the centre (Segment 2).

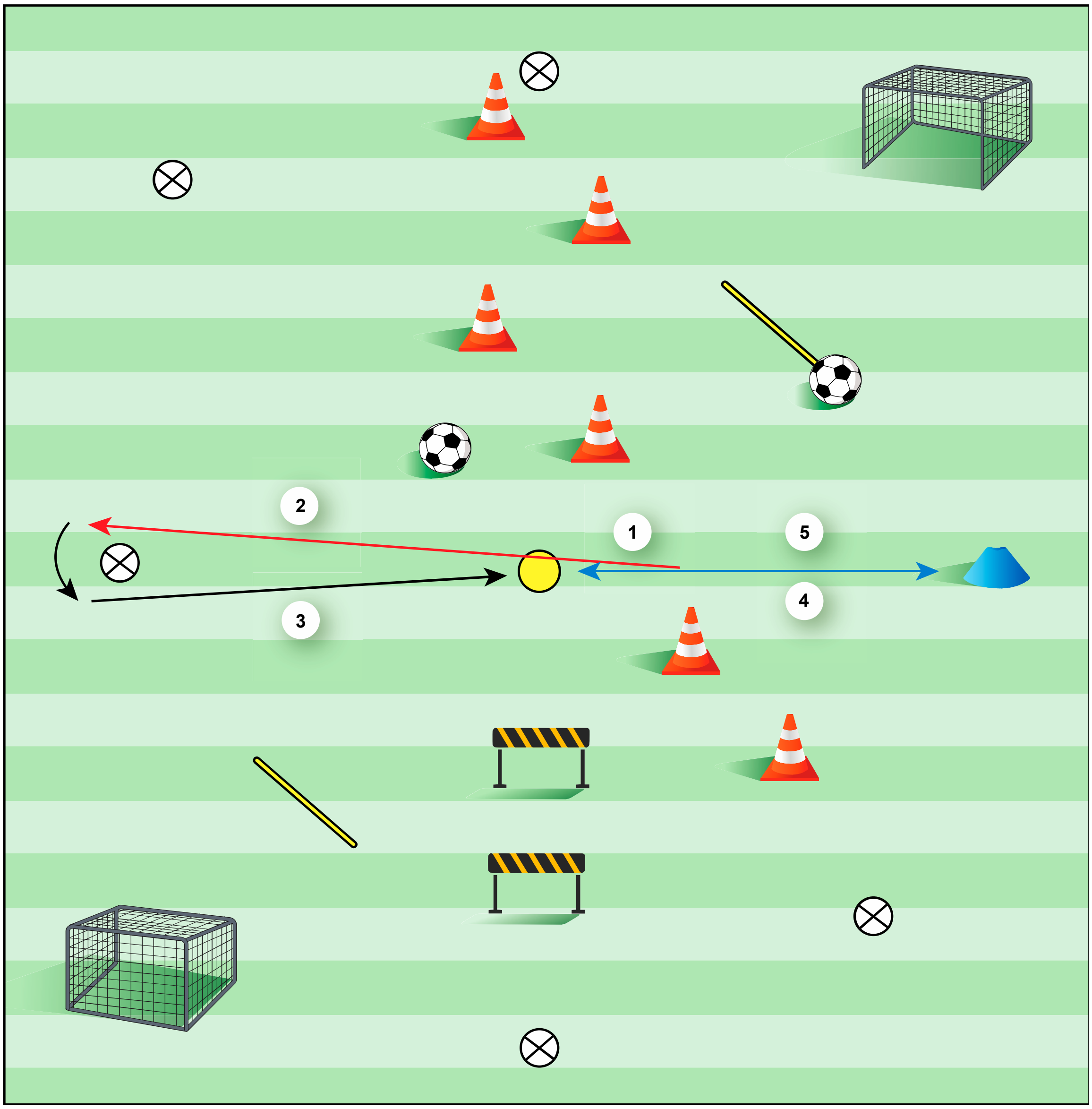

1	Test starting point
2	Forward-facing forward movement
3	Forward-facing back movement
4	Sideways movement to the right
5	Sideways movement to the left

Second diagonal: The participant must pass behind the central pole with a base and then perform a slalom movement to the pole with a base and turn, performing a zigzag movement stepping on the cones, until reaching the centre. From that moment, they must perform jumps and ball receptions with both feet until turning at the pole with a base, returning to the central pole with a base while performing jumps and receptions with one foot (changing foot at each jump) (Segment 4).

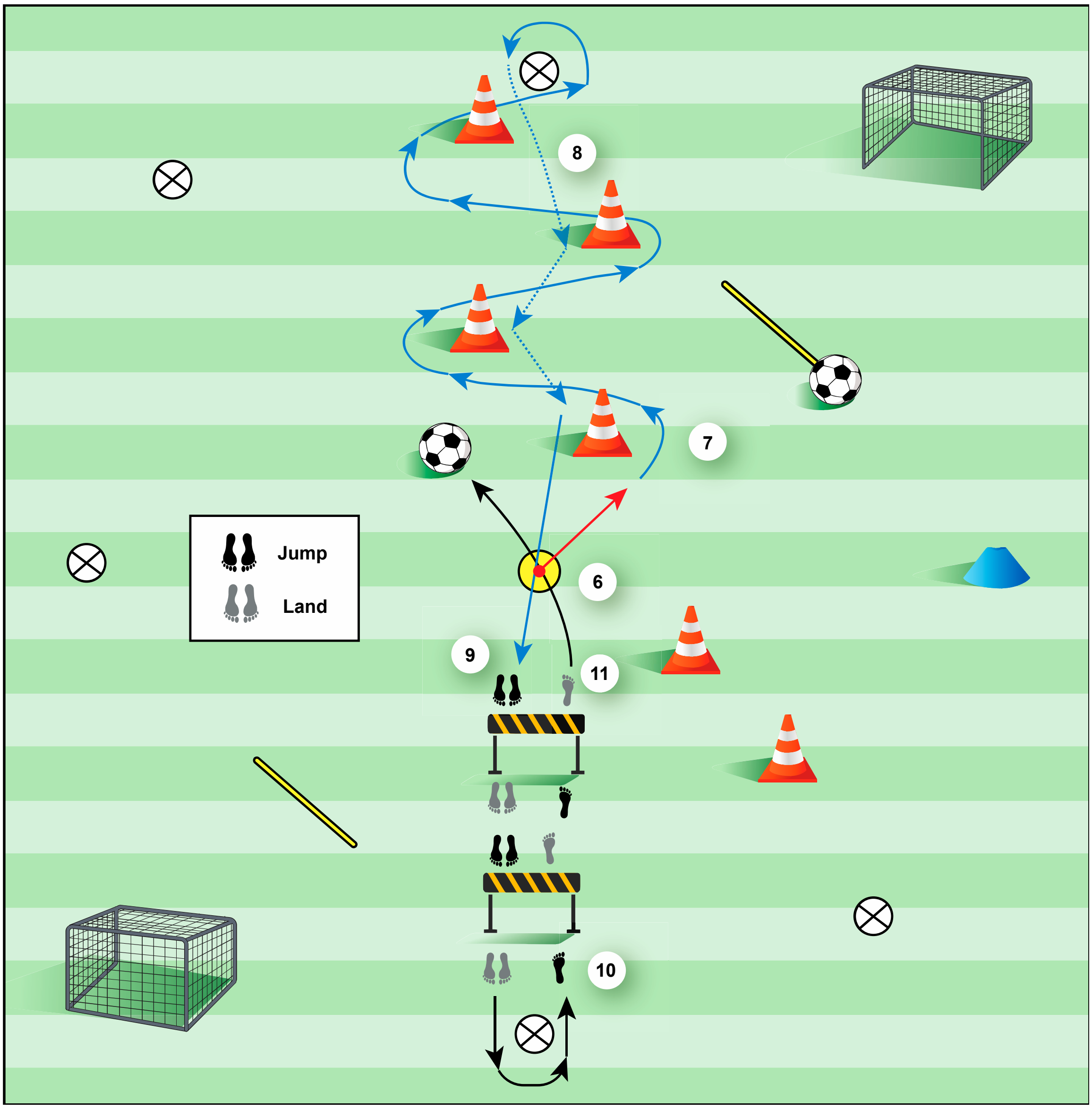

6	The Second diagonal starting point
7	Slalom movement
8	Zigzag movement stepping on the cones
9	Jump and ball reception with both feet
10	Jump and ball reception with one foot
11	Movement towards Segment 5 – Third diagonal

Third diagonal: The participant must pass behind the central pole with a base and then move towards Segment 5 to get the ball and dribble it with the **dominant** foot to the pole with a base and then turn, returning while dribbling with the **non-dominant foot** to the central pole (Segment 5). From that moment, the participant will face the cones (the participant must ensure that they do not knock over or move straight towards the second cone) to perform a zigzag dribble with the **dominant** foot to the pole with a base and then turn, returning while zigzag dribbling with the **non-dominant foot** to the central pole with a base (Segment 6).

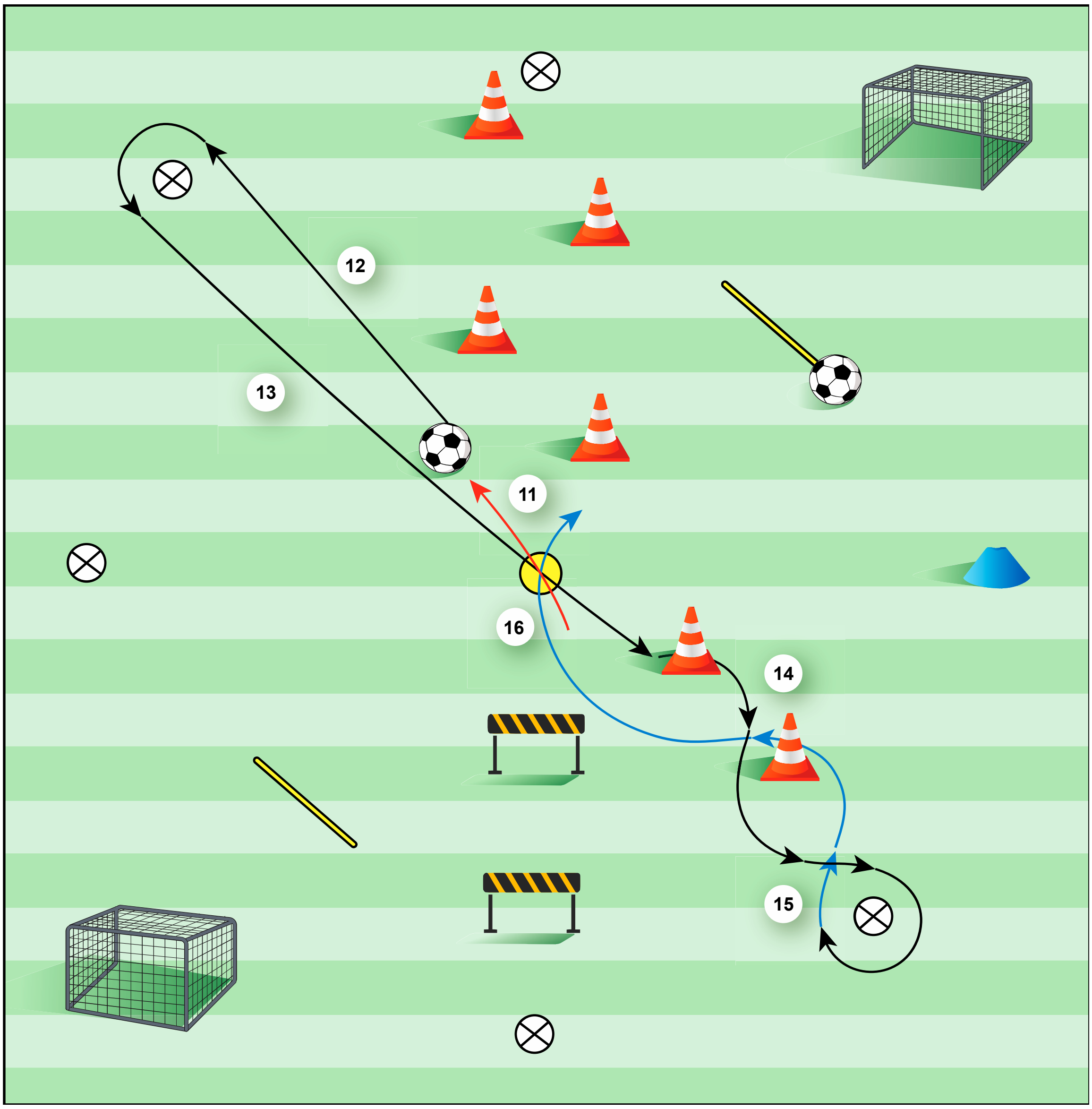

11	Third diagonal starting point – Movement towards Segment 5
12	Dribble with the dominant foot
13	Dribble with the **non-dominant** foot
14	Zigzag dribble with the dominant foot
15	Zigzag dribble with the **non-dominant** foot
16	End of third diagonal - Dribbling

Fourth diagonal: The participant must pass behind the central pole with a base and then move towards Segment 7 to reach the pole on the ground. They then shoot the ball with the **dominant** foot at the mini-goal. After that, they get the ball that is next to the mini-goal and dribble it freely to the pole of Segment 8. Once there, they shoot the ball with the **non-dominant** foot at the mini-goal, turn around, and run to the central pole with a base to finish the test.

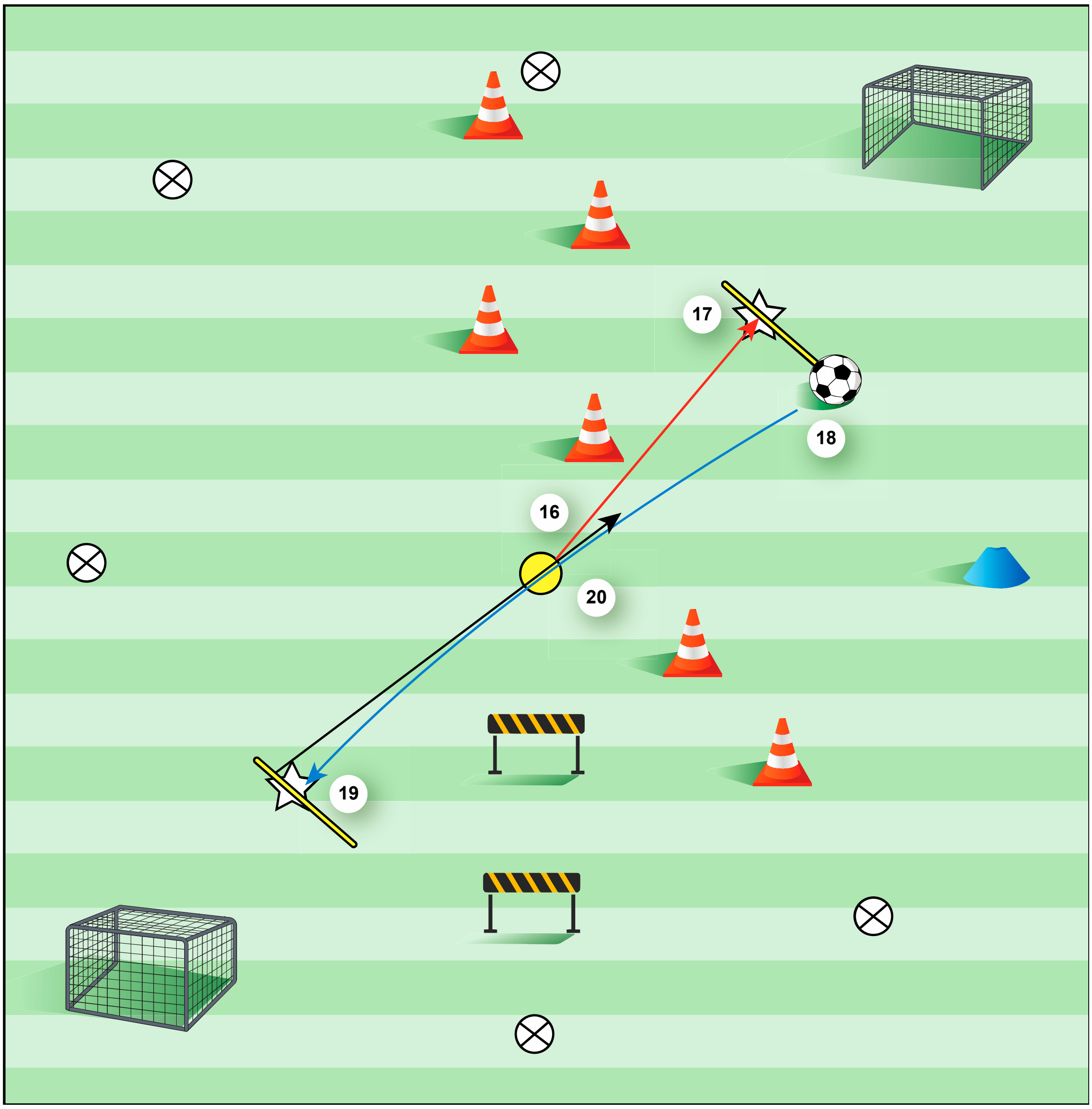

16 Fourth diagonal starting point, dribbling freely to point 17

17 Shoot the ball with the dominant foot (the ball must go into or hit the mini-goal)

18 Get the ball and dribble it freely to point 18

19 Shoot the ball with the **non-dominant** foot (the ball must go into or hit the mini-goal)

20 Return, running to point 20 and END of the ‘Rondo Test’.
